# Community-based trial of screening for *Chlamydia trachomatis *to prevent pelvic inflammatory disease: the POPI (prevention of pelvic infection) trial

**DOI:** 10.1186/1745-6215-9-73

**Published:** 2008-12-10

**Authors:** Pippa Oakeshott, Sally Kerry, Helen Atherton, Adamma Aghaizu, Sima Hay, David Taylor-Robinson, Ian Simms, Phillip Hay

**Affiliations:** 1 Division of Community Health Sciences, St George's, University of London, London, SW17 0RE, UK; 2 Department of Primary Care and Social Medicine, Imperial College, London, W2 1NY, UK; 3 Department of Health and Social Care Research, Kings College London, London, SE1 8WA, UK; 4 Department of Genitourinary Medicine and Communicable Diseases, Imperial College School of Medicine, St Mary's, London, W2 1NY, UK; 5 Health Protection Agency, Centre for Infections, London, NW9 5EQ, UK; 6 Department of Genitourinary Medicine, St George's Hospital, London, SW17 0RE, UK

## Abstract

**Background:**

Pelvic inflammatory disease (PID) is common and can lead to tubal factor infertility, ectopic pregnancy or chronic pelvic pain. Despite major UK government investment in the National Chlamydia Screening Programme, evidence of benefit remains controversial. The main aim of this trial was to investigate whether screening and treatment of chlamydial infection reduced the incidence of PID over 12 months. Secondary aims were to conduct exploratory studies of the role of bacterial vaginosis (BV) in the development of PID and of the natural history of chlamydial infection.

**Design:**

Randomised controlled trial with follow up after 12 months.

**Setting non-healthcare:**

Common rooms and lecture theatres at 20 universities and further education colleges in Greater London.

**Participants:**

2500 sexually active female students were asked to complete a questionnaire on sexual health and provide self-administered vaginal swabs and smears.

**Intervention:**

Vaginal swabs from intervention women were tested for chlamydia by polymerase chain reaction (PCR) and those infected referred for treatment. Vaginal swabs from control women were stored and analysed after a year. Vaginal smears were Gram stained and analysed for BV.

**Main outcome measure:**

Incidence of clinical PID over 12 months in intervention and control groups. Possible cases of PID will be identified from questionnaires and record searches. Confirmation of the diagnosis will be done by detailed review of medical records by three independent researchers blind to whether the woman is in intervention or control group.

**Trial registration:**

Clinical Trials NCT 00115388

## Background

Pelvic inflammatory disease (PID) is the most important preventable bacterial sexually transmitted infection in industrialised countries[1]. Studies in clinical settings suggest 15–50% of cases are caused by *Chlamydia trachomatis *infection[1]. After one episode of PID around 15% of women may become infertile, 10% suffer chronic pelvic pain and 10% of subsequent pregnancies may be ectopic which can be life threatening. The financial cost of treating PID and its sequelae, particularly tubal factor infertility, is considerable, quite apart from the emotional and physical costs to the woman.

There has been only one major trial in non-pregnant women of chlamydia screening to prevent PID[2]. This was conducted over 15 years ago in an American population using tests which have been superseded [2-4]. Before chlamydial screening became widespread in the UK, around 2006–2008, there was a window of opportunity to conduct a trial of community based screening using new nucleic acid amplification tests and non-invasive screening in a young, multiethnic, female student population[3].

In order to prevent chlamydia associated PID we also need to understand more about the natural history of chlamydial infection in women, and about the role of possible co-factors such as bacterial vaginosis (BV). Studies to date have been small[5] or cross-sectional[6], and a larger prospective study is needed[3]. We developed a special screening pack to test for chlamydia and BV using self-administered vaginal swabs[7]. The method was acceptable even during pregnancy and was used in our feasibility study in a student bar recruiting women to a trial of chlamydia screening[8]. The POPI (Prevention of Pelvic Infection) trial should provide new information on the effectiveness of screening for chlamydia, the possible role of bacterial vaginosis, and the natural history of chlamydial infection in the community.

## Methods/Design

### Primary aim

To investigate if screening young female students for chlamydial infection and treating those found to be infected reduces the incidence of PID in the subsequent 12 months.

### Secondary aims

We will conduct exploratory studies to investigate:

1. The role of BV at baseline on the development of PID over 12 months.

2. In women with untreated chlamydial infection, the proportion who have persistent infection or develop PID within 12 months.

3. The annual incidence of chlamydia infection in women without chlamydia at baseline who return follow up postal samples.

### Design

Randomised controlled trial over one year

**Figure 1:**
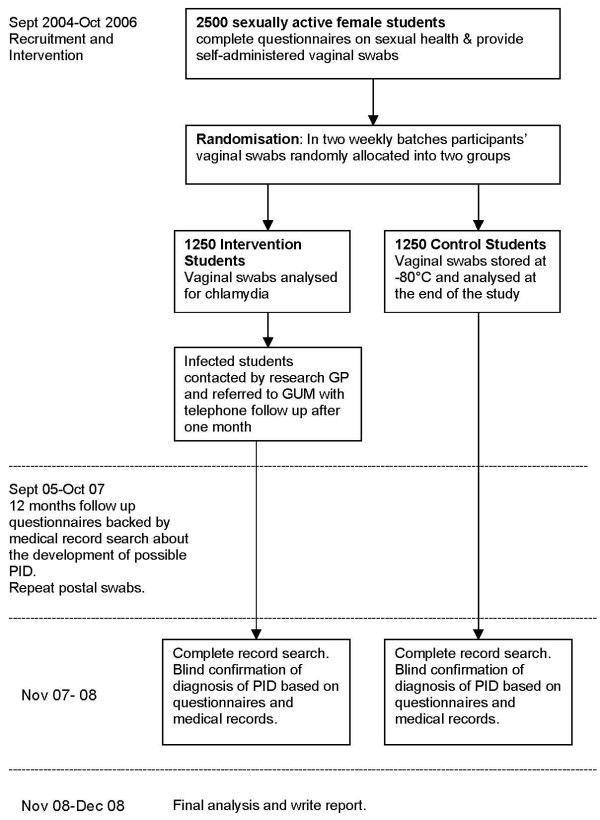
Design and timetable of trial of screening for chlamydia to prevent pelvic inflammatory disease (PID).

The flow of participants through the trial is shown in figure 1.

### Setting

Common rooms, bars and lecture theatres at 20 universities and further education colleges in Greater London.

Following agreement from the Students' Union Presidents, College Principals and welfare officers, posters about the study were displayed on the Students' Union website and notice boards. Recruitment was done by the research general practitioner (GP) and female research assistants assisted by trained peer recruiters as necessary.

### Participants

Sexually experienced female students aged ≤ 27 years were eligible for the study. Pregnant women, those who had never been sexually active, women aged >27, and women who had been tested for chlamydia in the past 3 months were excluded.

### Recruitment

Women considering taking part were given an information leaflet and invited to discuss the study with the research assistants or GP. Those who decided to participate were asked to sign a consent form, to complete a brief confidential questionnaire on sexual health and behaviour, to provide self-administered vaginal swabs in the nearest lavatory, and to consent to access to their medical records for the purposes of this study.

The baseline questionnaire asked about risk factors for chlamydial infection[9]: age, ethnicity, age at first sexual intercourse, number of sexual partners in the past year, contraceptive use; possible symptoms of infection: abnormal vaginal discharge, pelvic pain, dyspareunia, intermenstrual or post-coital bleeding; recent antibiotic treatment; history of sexually transmitted infection (STI); vaginal douching, smoking and quality of life (EQ-5D). To simplify recruitment, illustrated instructions on how to provide a self-administered vaginal swab were printed on specimen packs and displayed in the nearest lavatory, and specimen containers were prelabelled[7].

### Intervention

Within two weeks of recruitment participants were randomly allocated into two groups by researchers at St George's, University of London using random number tables starting with a number at any position and going consistently in any direction. Consecutive sealed specimen packs identified only by an ID number were taken out of the large carrier bag into which they had been put during recruitment sessions. If the random number ended in 0–4 the pack was put into the intervention pile; if it ended in 5–9 the pack was put into the control pile. Vaginal swabs were taken out of the specimen packs in the intervention pile and sent to the laboratory to be tested for chlamydial infection by transcription mediated amplification (TMA, Gen-Probe Incorporated San Diego, CA). Vaginal swabs from control women were stored at -80°C and analysed one year later. Infected women were contacted as soon as possible by the research GP and asked to attend their local Genitourinary Medicine (GUM) Clinic or GP for treatment and partner notification. One month later, the research GP telephoned the women again to answer any questions and to ensure that both they and their partner(s) had completed a course of treatment.

### Follow up after one year

A year after recruitment, participants were asked to complete a secure online questionnaire about PID symptoms during the previous 12 months. Those not responding or not providing an email address were sent the questionnaire by post backed up by telephone reminders. In order to maximise follow up, consent forms requested details of home and term time addresses and telephone numbers, email, mobile phone number, college course and GP address. The questionnaire asked about possible symptoms of, or treatment for, PID or other STI, any additional STI testing, and hospital and GP attendances in the past year. Women who reported no symptoms of possible PID on their questionnaire had no further follow up. For women who did not return a questionnaire and those who reported consulting a health professional for possible symptoms of PID we sent a questionnaire to their GPs. This asked GPs to search both their records and any hospital letters during the 12 month period after recruitment for possible symptoms of PID. In addition, all women who agreed on their follow up questionnaire to return a further swab were sent one in the post. These postal samples were analysed immediately and infected women referred for treatment.

### Further analysis of vaginal samples

A year after recruitment we analysed stored baseline vaginal swabs from women in the control group. Women whose swabs were positive for chlamydia were referred to GUM for retesting and treatment. Vaginal smears taken at baseline were Gram stained and examined for BV using Nugent's criteria[7]. Slides were read by two observers who were blinded to each other's results, to characteristics of participants and to outcome in terms of PID. Slides were categorised as positive (Nugent score 7–10), intermediate (4–6) or negative (0–3). Where the observers disagreed, the slide was reviewed until consensus was reached. There is little evidence that asymptomatic BV is harmful in non-pregnant women and no treatment was given. Participants worried about infection were advised to have a check up.

### Outcome measures

#### Primary outcome measure in the complete cohort

Incidence of clinical PID over 12 months in intervention and control groups.

Possible cases of PID will be identified from patient and GP 12 months questionnaires reporting pelvic infection, abdominal or pelvic pain or dyspareunia. For women who consulted a healthcare professional for these symptoms we will try to obtain clinical records including information on symptoms, findings on examination and laboratory results, excluding chlamydia results from trial samples. We will also try to obtain details on any woman reporting she had a laparoscopy in the follow up period. Since it would be unethical to demand a laparoscopic diagnosis of PID, as in the Scholes et al trial[2] we will use a modified version of Hager's clinical criteria[10] where details are provided in the medical records: lower abdominal pain, adnexal tenderness and tenderness on cervical motion. If available we will also include the results of concurrent microbiological tests and clinical data on fever >38°, leucocytosis >10,000, purulent material or pelvic abscess. To reduce bias, confirmation of the diagnosis will be done by review of all data (anonymised questionnaires and clinical records) by two independent GUM physicians (with review by a third where they disagree) who will be blind as to whether the patient is in intervention or control group.

#### Secondary outcome measures

We will conduct exploratory studies to investigate:

1. The role of BV at baseline on the development of PID over 12 months.

2. In women with untreated chlamydial infection, the proportion who have persistent infection or develop PID within 12 months.

3. The annual incidence of chlamydia infection in women without chlamydia at baseline who return follow up postal samples,

#### Blinding

The GU physicians will be blind to group assignment. They will categorise cases of PID as probable, possible or not PID, for example if subsequent laparoscopy suggests the pain is due to endometriosis. The research assistant who selected the questionnaires and anonymised the medical records for assessment for possible PID was also blind to study group assignment.

All participants were blind except those in the intervention group whose baseline samples were positive for chlamydia and who were referred for treatment. It is possible that the principle investigator who contacted women to tell them they were chlamydia positive might remember some names when assisting with obtaining medical records for follow up 1–2 years later. There was a change of research assistant between recruitment and final follow up. The research assistant doing the final follow up for PID diagnosis was not involved in randomization or testing and treatment of chlamydia positives. Both she and the PI had access to group allocation but avoided looking at this during follow up.

### Sample size calculations

Assuming a 2% incidence of PID, a sample size of 4122 women would allow a relative risk (RR) of 0.48 to be detected with 80% power using a 5% significance level. This was based on data from the Scholes et al trial[2] where the rate of chlamydia was 7%. Assuming 20% of women were lost to follow up, we would have needed to recruit 5000 women.

However we had great difficulties with recruitment, and a publication in 2004 suggested a higher rate of PID[11] enabling us to revise down our sample size calculations. If we assume a 3% incidence of PID in the control group[11,12], we would need a sample of 2274 women to detect a RR of 0.44% with 80% power and 5% significance. Details on the consent form provided up to 7 potential contact methods for follow up, and we achieved 92% follow up after 8 months in the feasibility study[8]. To reduce bias we aimed to keep loss to follow up at 12 months below 10%. Assuming 10% loss to follow up we needed to recruit 2501 women.

### Analysis Plan

For the primary analysis, we will estimate the relative risk of developing PID in the 12 months after recruitment in the intervention group compared with the control.

In secondary analyses, we will conduct an exploratory logistic regression to adjust the RR of development of PID for the presence of baseline BV.

We will conduct further exploratory analyses to assess:

In women with untreated chlamydial infection, the proportion (95%CI) who have persistent infection or develop PID within 12 months.

The annual incidence (95%CI) of chlamydia infection in women without chlamydia at baseline who return follow up postal samples.

### Ethical issues

The POPI trial was approved by Wandsworth Research Ethics Committee in 2003 (Ref 03.0054). The main ethical issue was that samples from control women were stored and not tested for chlamydia for 12 months. This was clearly explained to the women before they participated and repeated on the information sheet and consent form (attached). In addition an extra copy of the information sheet was posted to their home address. The patient information leaflet (please see additional file 1) stated:

"What are the risks of taking part? When you sign the consent form you should note that your vaginal sample will be tested for a sexually transmitted infection called chlamydia. However the test may not be done for a whole year. You could have chlamydial infection without experiencing any symptoms. This could lead to pelvic inflammatory disease with a risk that you have chronic pelvic pain, become infertile, or that if you become pregnant that you have an ectopic pregnancy (a pregnancy in a fallopian tube which can be fatal). *This is why if you could have been at risk of infection, or have any symptoms which could be due to a sexually transmitted infection, it is vital that you have a check up at a genitourinary clinic even if you are participating in the study"*.

## Discussion

Recruiting young women to a trial of chlamydia screening proved much harder than anticipated. We developed new techniques to market the trial to students, particularly recruiting from lectures and offering lollipops[13,14]. To achieve our sample size we also had to extend recruitment to 20 educational institutions and to prolong the recruitment period to 2 years (from September 2004 to October 2006.) For the first 4 months of recruitment, peer recruiters completed response rate forms. Of 2323 women approached 41% (956) were ineligible: 41% (n = 390) had never had sexual intercourse, 24% were aged <16 (n = 19) or >27 years (n = 206), and 14% (n = 136) had been tested for chlamydia in the previous 3 months. The response rate in those eligible was 32% (440/1367). Reasons for refusal (n = 927) included "not interested" 35%, "too busy" 31%, "don't want to provide swab" 13%. Recruitment rates seemed to improve later in the trial and were over 60% in the last three sessions[14].

## Conclusion

The trial will be the first to show if screening for chlamydial infection can reduce the incidence of PID in a British population. It will also show if non-invasive screening of high risk, difficult to access groups such as women aged <20 and those from black ethnic minorities is feasible outside health care settings. Over 40% of young people in the UK now attend higher education adding to the generalisability of the results. If the intervention is effective, extending such screening programmes to the community should help to reduce the burden of PID. Finally by providing new information on both the effectiveness of screening and on the natural history of chlamydial infection and the influence of BV, the study will contribute to the evidence base for the UK National Strategy for Sexual Health. The trial will report in December 2008.

## Abbreviations

PID: pelvic inflammatory disease; POPI trial: Prevention of Pelvic Infection trial; BV: bacterial vaginosis; TMA: transcription mediated amplification; RR: relative risk; GP: general practitioner; STI: sexually transmitted infection; GUM: Genitourinary Medicine.

## Competing interests

The authors declare that they have no competing interests.

## Authors' contributions

PO, PEH and SK conceived and designed the study with help from IS, DTR, SH and HA. PO, SK, AA and HA drafted the manuscript for submission to Trials. All authors read and approved the final manuscript.

## Supplementary Material

Additional File 1 Appendix. Patient information leaflet. Click here for file
