# Supplementary material for: Community-based trial of screening for Chlamydia trachomatis to prevent pelvic inflammatory disease: the POPI (prevention of pelvic infection) trial
Source: Trials. 2008 Dec 10;9:73. doi: 10.1186/1745-6215-9-73 (PMC2614935; doi:10.1186/1745-6215-9-73)
Supplement: Additional File 1 — Appendix. Patient information leaflet. [file 1745-6215-9-73-S1.doc]

Appendix


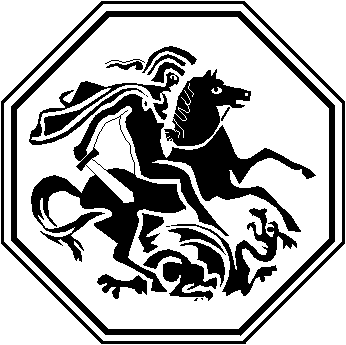
 Department of Community Health Sciences

St George's Hospital Medical School

University of London

Hunter Wing

Cranmer Terrace

London SW17 0RE

020872515715

**March 05**

## PATIENT INFORMATION SHEET

#

# Project title: POPI Study – Prevention of Pelvic Infection

# 2. Invitation to participate in a research study on women’s health

# You are being invited to take part in a research study. Before you decide it is important for you to understand why the research is being done and what it will involve. Please take time to read the following information carefully and discuss it with friends. Ask us if there is anything that is not clear to you. *Thank you for reading this.*

# What is the purpose of the study?

# Treatment of a common sexually transmitted disease called chlamydial infection may prevent infection of the womb and fallopian tubes – pelvic inflammatory disease or PID. The main aim of this study is to see if screening and treating young women for chlamydial infection can reduce the risk of PID. A second aim is to see if PID is associated with possible co-factors such as a mild vaginal infection called bacterial vaginosis.

# 4. Why have I been chosen?

# We are asking female students to help us if they are aged 27 or younger and can provide an address, phone no or email where we can contact them in a year’s time.

# 5. Do I have to take part?

# It is up to you to decide whether or not to take part. If you do decide to take part you will be given this information sheet to keep and be asked to sign a consent form. If you decide to take part you are still free to withdraw at any time and without giving a reason. As the study is investigating sexually transmitted infections, only students who have had penetrative sexual intercourse with a man should consider taking part.

# 6. What will happen to me if I take part?

You will be asked to complete a short confidential questionnaire on sexual health and to use two small cotton buds to provide vaginal samples. You will be given simple instructions on how to provide the samples in the nearest lavatory.

We will ask for your address, mobile number and email address so we can send you another questionnaire and ask for repeat postal samples after one year. We also need your permission to contact your GP and examine your medical records including any genitourinary medicine and hospital records at the end of the study.

In order to see if chlamydia screening prevents PID, around 50% of participants will have their samples tested for chlamydia within about 6 weeks of being recruited.

If the test shows you have chlamydial infection, we will contact you and arrange for you and your partner to attend The Courtyard Genitourinary Medicine Clinic at St George’s Hospital where you will be offered antibiotics to clear up the infection.

The chlamydia samples from the remaining 50% of participants will not be checked for infection until the end of the study in a year’s time. *Therefore it is very important that you understand that if you are worried that you may have been exposed to risk of a sexually transmitted infection you should get checked at a genitourinary clinic.*

eg The Courtyard Clinic, St George’s Hospital, Tooting, London SW17 Tel 020 8725 3353. Alternatively ring NHS Direct on 0845 4647.

**7. What are the risks of taking part?**

**When you sign the consent form you should note that your vaginal sample will be tested for a sexually transmitted infection called chlamydia. However the test may not be done for a whole year. You could have chlamydial infection without experiencing any symptoms. This could lead to pelvic inflammatory disease with a risk that you have chronic pelvic pain, become infertile, or that if you become pregnant that you have an ectopic pregnancy (a pregnancy in a fallopian tube which can be fatal). *This is why if you could have been at risk of infection, or have any symptoms which could be due to a sexually transmitted infection, it is vital that you have a check up at a genitourinary clinic even if you are participating in the study.***

**8. What are the possible benefits of taking part?**

At the end of the study after one year we will test the remaining chlamydia samples. If we find you were infected we will contact you and recommend that you attend the genitourinary clinic at St George’s Hospital for further testing and treatment.

**9. What if something goes wrong?**

If you have chlamydial infection, we will arrange for you to be treated by doctors at the genitourinary clinic so you will be able to discuss any problems with them in the usual way. In addition the routine NHS complaints mechanisms will be available to you.

**10. Will my taking part be kept confidential?**

**All information which is collected about you during the course of the research will be kept strictly confidential. Any information about you which leaves the research centre will have your name and address removed so that you cannot be recognised from it. If the test shows you have chlamydial infection we will contact you to arrange treatment. We will not inform your GP or anyone else without your consent.**

**11. What will happen to the results of the research study?**

The results of the study will be published in a scientific journal within 3-5 years. A copy of the published results will be available from Dr Oakeshott (details below).

**12. Contact for further information**

If you have any questions the research team will be happy to answer them. Please feel free to contact Dr Pippa Oakeshott and team in The Department of Community Health Sciences, St George’s Hospital Medical School, London SW17 0RE.

Telephone 0208 725 0153 / 5715 Email: oakeshot@sghms.ac.uk

| **CONSENT FORM FOR POPI STUDY- Prevention of Pelvic Infection** |
| --- |

**Name of Researcher: Dr Pippa Oakeshott *Please initial box***

1. **I confirm that I have read and understand the information sheet dated March 2005 for the above**

**study and have had the opportunity to ask questions.**

1. **I understand that my participation is voluntary and that I am free to withdraw at any time, without**

**giving any reason, without my medical care or legal rights being affected.**

1. **I understand that sections of any of my medical notes may be looked at by responsible individuals from**

**St George’s Hospital Medical School where it is relevant to my taking part in research. I give permission for the researchers to have access to my GP, hospital and / or genitourinary medicine records.**

1. **I understand that the vaginal samples which I am to provide today will be tested for a sexually transmitted infection called chlamydia, but that this test may not be done for a year from now. I also understand that I could have chlamydial infection without knowing about it, and that this could result in pelvic inflammatory disease, chronic pelvic pain, infertility or ectopic pregnancy. I understand that if I have been at risk of the infection it is vital that I get checked at a genitourinary clinic and *I accept that it is my responsibility to do this.***

**5. I agree to take part in the above study.**

**I agree to provide vaginal samples now and to complete a short, confidential questionnaire on sexual health. I understand that I will be asked to provide another sample and to complete a further questionnaire which will be posted to me after 12 months.**

**Name of Participant in capitals Date Signature**

**Address and postcode**

**_________________________________________________________________________________________**

**Date of birth______________________________ Email __________________________________________**

**Mobile and Tel no_________________________________________________________________________**

**Home address if different from above _______________________________________________________**

**_________________________________________________________________________________________**

**GP name, address (and telephone no if known)_________________________________________________**

**_________________________________________________________________________________________**

**Course and College_________________________________________________________________________**

**_______ __**

**Researcher Date Signature**
